# Rosiglitazone promotes glucose metabolism of GIFT tilapia based on the PI3K/Akt signaling pathway

**DOI:** 10.14814/phy2.14765

**Published:** 2021-03-02

**Authors:** Dong‐Yan Guan, Hui‐Wen Sun, Ji‐Ting Wang, Zhen Wang, Yang Li, Hao‐Jun Han, Xiang Li, Ting‐Ting Fang

**Affiliations:** ^1^ Shandong Provincial Key Lab. of Animal Biotechnology and Disease Control and Prevention Lab of Aquatic Animal Nutrition & Environmental Health Shandong Agricultural University Taian City Shandong Province China

**Keywords:** GIFT tilapia, glucose metabolism, growth, PI3K/Akt, rosiglitazone

## Abstract

The study aimed to explore the effects of rosiglitazone on glucose metabolism of GIFT tilapia based on the PI3K/Akt signaling pathway. The experiment was divided into five groups: normal starch group (32%, LC), high starch group (53%, HC), high starch +rosiglitazone group 1 (10 mg/kg, R1), high starch + rosiglitazone group 2 (20 mg/kg, R2), and high starch + rosiglitazone group 3 (30 mg/kg, R3). The results showed that a high starch diet supplemented with 10–20 mg/kg rosiglitazone had a better specific growth rate and protein efficiency that was beneficial for the growth of the tilapia. Rosiglitazone had no significant effect on the contents of crude lipid, crude protein, crude ash, and moisture of the whole fish body (*p* > 0.05). The contents of triglycerides and total cholesterol in the R1, R2, and R3 groups were lower than those in the HC group. The levels of glutamic oxaloacetic transaminase (GOT) and glutamic pyruvic transaminase (GPT) in R1 and R2 groups were significantly lower than those in the HC groups (*p* < 0.05). However, the GOT and GPT levels in the R3 groups were significantly higher than those in the R1 and R2 groups (*p* < 0.05). With an increase in the rosiglitazone concentration, the contents of serum glucose, insulin, and hepatic glycogen in the R1, R2, and R3 groups decreased gradually. Meanwhile, the muscle glycogen content in the R1, R2, and R3 groups increased gradually. The mRNA expression of the IRS‐1, PI3K, GLUT‐4, and Akt proteins in the R1, R2, and R3 groups was significantly higher than that in the HC group (*p* < 0.05). Compared with the HC group, the expression of the GSK‐3 mRNA in the R1, R2, and R3 groups was significantly reduced (*p* < 0.05). The protein expression of p‐Akt in the R1 and R2 groups was higher than that in the HC group (*p* > 0.05). The protein expression of p‐GSK‐3β in the R1 and R2 groups was significantly higher than that in the HC group (*p* < 0.05). In conclusion, a high starch diet supplemented with rosiglitazone can improve growth, enhance the serum biochemical indices, and increase the muscle glycogen content in the GIFT tilapia. It benefits in upregulating the IRS‐1, PI3K, and GLUT‐4 mRNA levels in the skeletal muscle and promotes glucose uptake. Meanwhile, the phosphorylation of Akt and GSK‐3β increased significantly and resulted in the inactivation of GSK‐3β and alleviation of insulin resistance.

## Graphical abstract

A high starch diet supplemented with rosiglitazone can improve the growth performance, reduce the serum glucose and insulin levels, improve the blood biochemical indices, and increase the muscle glycogen content. Rosiglitazone upregulates the mRNA levels of IRS‐1, PI3K, and GLUT‐4 in the skeletal muscle, increases the insulin sensitivity, and promotes glucose uptake.

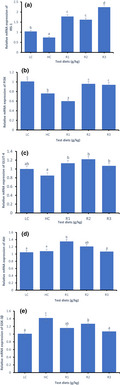

## 1 INTRODUCTION

An appropriate amount of carbohydrates in the fish feed can be utilized as an energy source for decomposition and energy supply and can effectively reduce the amount of protein used (Helland & Grisdale‐Helland, 1998). Moreover, it can improve the utilization rate of feed carbohydrates, increase the tolerance of fish to carbohydrate, reduce the feed cost, and improve the economic benefits (Han, 2018). In addition, appropriate carbohydrates can reduce water pollution and the eutrophication caused by protein decomposition, increase ATP synthesis in the fish body, and promote fish protein synthesis (Tan et al., 2007). However, compared with terrestrial organisms, fish are not tolerant of carbohydrates (Wilson, 1994). When the carbohydrate content in the feed is too high, the health status of the fish is severely damaged, and growth and feed utilization are negatively affected (Hemre et al., 1995), leading to the decline of stress resistance of fish (Shen, 2018). High carbohydrate content increases fat accumulation in the liver thereby forming fatty liver (Lie et al., 1988); this may damage the liver and even cause the death of the fish (Dixon & Hilton, 2006). Therefore, improving the utilization efficiency of carbohydrates in fish has become a topic of debate. Simultaneously, the study of the sugar metabolism mechanism of fish is of great practical significance in aquaculture, and reasonable ways to improve carbohydrate utilization efficiency need to be explored.

Insulin is the core substance of glucose metabolism, and it can strictly control the utilization of glucose (Mommsen et al., 2001). Insulin and insulin‐like growth factor (IGF‐I) bind to each other's receptors and exert their effector functions through a common information transmission system (Shi et al., 2012). Currently, there are at least two known insulin signal transduction pathways: the phosphatidylinositol 3‐kinase (PI3K) pathway and the mitogen‐activated protein kinase (MAPK) pathway (Cohen et al., 1997). The insulin receptor mediates some key functions through the PI3K/Akt pathway such as regulating the proliferation and differentiation of skeletal muscle myoblasts and modulating the absorption of sugar and amino acids in tissues. The PI3K/Akt pathway mediates the translocation of the glucose transporter 4 in response to insulin, promotes glucose uptake by the peripheral cells such as the skeletal muscle cells, and thus regulates the glucose metabolism (du Toit‐Kohn et al., 2009). Insulin receptor‐mediated MAPK and PI3K/Akt signal transduction pathways exert completely different effects on the mammalian skeletal muscle (Liu et al., 2000). However, very little information is known about the insulin receptor‐mediated signal transduction pathway in fish. Pozios et al. (2001) found that IGF‐1 stimulates embryonic cell proliferation in the zebrafish by activating the MAPK and PI3K signaling pathways. In the rainbow trout, Castillo et al. (2006) found that insulin receptors in the muscle cells at different culture stages mediate different signal transduction pathways of MAPK and PI3K/Akt. In conclusion, the role of fish insulin receptor‐mediated signal transduction in insulin function (such as regulating glucose metabolism) is worthy of further study. In this study, the genes related to the insulin signal transduction pathway were studied to find an effective way to improve the utilization rate of carbohydrate in fish.

Rosiglitazone is a type of human glucose metabolism regulator that can increase insulin sensitivity, improve the ability of insulin binding to its receptor, regulate glucose metabolism in the human body, and effectively reduce fasting and postprandial blood glucose levels. However, there are few reports on the application of glucose metabolism regulators in fish. At present, humans and rodents are the main models of human diabetes research. However, some fish have novel and unique advantages compared with the classical models as they are important and innovative model system for diabetes research. Most studies on fish glucose metabolism have been performed with zebrafish (*Danio rerio*) and rainbow trout (*Oncorhynchus mykiss*). The advantages of evolution can help study the cases of natural insulin resistance in fish (Elo et al., 2007; Krishnan & Rohner, 2019). However, no investigations have been carried out on tilapia. This experiment was conducted to investigate the effects of rosiglitazone on the growth and the PI3K/Akt signal transduction pathway in the skeletal muscle of GIFT (Genetic Improvement of Farmed Tilapia) tilapia (*Oreochromis niloticus*) to understand the regulation mechanism of carbohydrate metabolism in fish and to provide a new perspective to utilize carbohydrate effectively and save feed protein. The GIFT tilapia were reared on a high carbohydrate diet to improve the tolerance and utilization rate of carbohydrate in fish. The results of this study can prove to be of great significance in promoting the sustainable development of aquaculture and alleviating the shortage of protein feed resources.

## 2 MATERIALS AND METHODS

### 2.1 Ethics statement

This study was conducted at College of Animal Science, Shandong Agricultural University, China, and was approved by the scientific ethics committee of Shandong Agricultural University. All experimental protocols and methods were carried out in accordance with the relevant national, Shandong agricultural university and local laws and regulations.

### 2.2 Experimental diets and design

Tilapia were fed a normal starch diet (LC, positive control containing 320 g/kg starch), high starch diet (HC, negative control containing 530 g/kg starch), and the high starch diet supplemented with 10, 20, or 30 mg/kg rosiglitazone (R1, R2, R3, respectively). The pellet feed was processed as described by Guan et al. (2019). Before preparing the feed, the raw materials were crushed and allowed to pass the No. 60 mesh (250 µm) sieve. All crushed feed materials were mixed evenly according to the feed formula provided in Table 1. Later, corn oil was added, the tiny oil particles were rubbed by hand, and finally, distilled water was added to form a hard mass from the powder feed. The wet mash was extruded into a 2‐mm diameter particle strip with a small‐sized flat die pelletizer, dried naturally, and stored at 4°C.

**TABLE 1:** Feed composition and nutrient components of the experimental diets (g/kg).

Ingredient	LC	HC	Rosiglitazone level (mg/kg)		
			R1(10)	R2(20)	R3(30)
Fish meal	300.0	300.0	300.0	300.0	300.0
Soybean meal	210.0	120.0	120.0	120.0	120.0
α‐Starch	320.0	530.0	530.0	530.0	530.0
Carboxymethyl cellulose	70.0	0.0	0.0	0.0	0.0
Corn oil	72.0	22.0	22.0	22.0	22.0
Calcium dihydrogen phosphate	20.0	20.0	20.0	20.0	20.0
Vitamin‐premix^a^	2.0	2.0	2.0	2.0	2.0
Mineral‐premix^b^	2.0	2.0	2.0	2.0	2.0
Choline chloride (50%)	4.0	4.0	4.0	4.0	4.0
Rosiglitazone	0.0	0.0	0.01	0.02	0.03
Total (kg)	1000.0	1000.0	1000.0	1000.0	1000.0
Nutrition levels (calculated value)					
Crude protein (%)	28.26	24.12	24.12	24.12	24.12
Crude lipid (%)	10.30	5.40	5.40	5.40	5.40
Gross energy (KJ/g)	17.67	17.76	17.76	17.76	17.76
Total carbohydrate (%)	33.68	53.96	53.96	53.96	53.96
Total phosphorous (%)	1.10	1.10	1.10	1.10	1.10

^a^ Vitamin premix (mg/kg diet): retinol acetate 30 mg; cholecalciferol 5 mg; alpha‐tocopherol 60 mg; ascorbic acid 600 mg; vitamin K3 7 mg; thiamin 20 mg; riboflavin 20 mg; pyridoxine HCL 12 mg; vitamin B12 0.05 mg; inositol 100 mg; pantothenic acid 50 mg; niacin acid 35 mg; folic acid 8 mg; biotin 0.06 mg.

^b^ Mineral premix (mg or g/kg diet): KI (1%) 60 mg; CoCl_2_·6H2O (1%) 7 mg; CuSO_4_·5H_2_O 20 mg; FeSO_4_·H_2_O 300 mg; ZnSO_4_·H_2_O 200 mg; MnSO_4_·H_2_O 60 mg; Na_2_SeO_3_·5H_2_O (1%) 60 mg; MgSO_4_·7H_2_O 2600 mg.

### 2.3 Fish and growth experiment

The fish were domesticated in a controlled water circulation system for 2 weeks and fed the basic diet before beginning the experiments. A total of 750 fish of homogenous size (80.33 ± 0.68 g) were randomly distributed into 25 tanks (400 L, 30 fish per tank) for five types of dietary treatments; each diet type had five replicates. In the feeding experiment that spanned 50 days, the food was supplied according to the fish requirements; the uneaten feed was gathered after every meal by plastic nets, dried, and weighed. The fish were fed thrice a day. Water quality parameters included temperature 26.5 ± 3.5°C, pH 7.3 ± 0.3, dissolved oxygen 5.8 ± 0.4 mg/L, ammonia‐N less than 0.05 mg/L, and nitrite‐N less than 0.03 mg/L. After the feeding experiment, the fish were maintained under fasting conditions for 24 h. The total weight of each aquarium was weighed, the feed intake of each tank was recorded, and the growth performance index of each group of fish was calculated.

### 2.4 Sample collection and chemical analysis

At the end of the feeding experiment, five fish were randomly selected from each aquarium and frozen (−20°C) for the analysis of whole body composition. Another batch of five fish from was selected from each aquarium for serum biochemical index analysis. MS‐222 (solution concentration at 100 mg/L) was used to anesthetize the sample fish. Blood was collected from the caudal vein of the fish and centrifuged at 4000 *g* at 4°C for 10 min; the separated serum was frozen at −80°C for further analysis. The next batch of five fish from each tank was anesthetized and then dissected to obtain the dorsal muscle and hepatopancreas samples. Each batch of the five fish was collected in a bag and labeled with the sample number and date. These samples were immediately stored at −80°C for further use.

The contents of protein, serum glucose, total cholesterol, triglycerides, and activities of glutamic oxaloacetic transaminase (GOT) and glutamic pyruvic transaminase (GPT) in the serum were determined by the colorimetric enzymatic method. The insulin and liver/muscle glycogen were measured by the peroxidase assay and colorimetry, respectively. All indices were tested using the assay kit from Nanjing Jiancheng Bioengineering Co., Ltd (China), and operated according to the manufactures's instructions. The contents of dry matter, crude protein, crude lipid, and ash in the whole fish body were analyzed according to the AOAC (2000) method.

### 2.5 Gene expression analysis of PI3K, IRS‐1, Akt, GSK, and GLUT‐4 in the dorsal muscle

#### 2.5.1 RNA extraction and cDNA synthesis

A small piece of the dorsal muscle (80 mg) of the test fish was ground into a fine powder with a mortar and pestle and placed in liquid nitrogen. The frozen powder was transferred to a 1.5 ml Eppendorf tube, and 1 ml of TRIzol reagent was added. Total RNA was isolated with Trizol according to the manufacturer's instructions. The content of RNA in the samples was determined by spectrophotometry; the integrity (quality) of the samples was detected by denaturing gel electrophoresis (1% agarose gel). The 260/280 nm absorbance ratio of all samples was between 1.8 and 2.0, indicating the purity of the RNA samples.

The first‐strand cDNA was synthesized from 1 μg of RNA using the PrimeScript RT Reagent Kit with the gDNA Eraser (Perfect Real Time; TaKaRa Biotechnology, Dalian, China) following the manufacturer's protocol. The synthesized cDNA was diluted 10‐fold for the following qRT‐PCR analysis.

#### 2.5.2 Expression analysis by quantitative PCR

The gene expression levels were measured by quantitative real‐time PCR (qPCR) on the prepared cDNA using the ABI7500 Real‐Time PCR system (Applied Biosystems, Carlsbad, CA, USA) with the SYBR Premix Ex Taq (Takara Bio Inc. China) detection reagents.

Gene‐specific primer sequences are given in Table 2. All PCR reactions were run in duplicate and normalized using (Livak & Schmittgen, 2001) the β‐actin gene as a reference.

**TABLE 2:** Nucleotide sequences of the primers for real‐time qRT‐PCR amplification.

Primer pairs	Sequence (5’→3’)	GenBank no. (gene symbol)
PI3K‐F	CCAAAACCACTACCTCTGCG	XM‐019358208.2
PI3K‐R	TCGTTCTTCATCAATGCCAA	XM‐019358208.2
IRS‐1‐F	GCCTCCTTACCTCCTATG	XM‐019355166.2
IRS‐1‐R	GGCTCGTGCTTCTTGACA	XM‐019355166.2
Akt‐F	GACCACAACCTCCCACCG	XM‐003441588.5
Akt‐R	TCGCATCCATTCCTCCCT	XM‐003441588.5
GSK‐F	CTGGTGACAGCGGAGTGGAC	XM‐019353012.2
GSK‐R	GGCAGGGATGAGTATGGAGG	XM‐019353012.2
GLUT‐4‐F	TCATCATCGCCATCCTCC	JN900493
GLUT‐4‐R	CGACCCGTCCTCTCTACC	JN900493
β‐actin‐F	TGACCTCACAGACTACCTCATG	KJ126772.1
β‐actin‐R	GGCAACGGAACCTCTCATTG	KJ126772.1

F: upstream primer R: downstream primer.

### 2.6 Western blot analysis

The RIPA cell lysate and PMSF protease inhibitor were mixed in the ratio of 99:1 to prepare the substrate working fluid solution. Into a 1.5‐ml centrifuge tube, 800 µl of the working fluid was added and precooled in advance. The muscle sample (50 mg) was taken and immediately added into a precooled mortar, liquid nitrogen was added while grinding until the sample formed a powder. The ground sample was rapidly transferred into the 1.5‐ml centrifuge tube that contains the precooled working fluid. After complete vibration and mixing, the ultrasonic crusher was used thrice for crushing. The above samples were centrifuged at 4°C at 12,000 *g* for 10 min, and the supernatant was withdrawn to determine the protein concentration.

The protein concentration of the samples was determined by the BCA method. The 5 X protein loading buffer was added to each sample (40 μg) in the ratio of 4:1, heated at 100°C for 5 min and then electrophoresed. After electrophoresis, the separated proteins were transferred to a polyvinylidene fluoride (PVDF) membrane (150 mA, 4°C, 1.5 h). After membrane transfer, the PVDF membrane was removed and washed with TBST buffer for 2 min. It was then transferred to a glass dish that contains the sealing liquid and placed on a shaking table for 1.5 h.

After sealing, the PVDF membrane was washed thrice with the TBST buffer solution for 8 min each. This membrane was then placed in the corresponding primary antibody (the dilution ratio of GAPDH was 1:3000, the dilution ratio of p‐Akt was 1:2000, and the dilution ratio of p‐GSK was 1:1000) and incubated overnight at 4°C. The incubated PVDF membrane was washed thrice with the TBST buffer for 8 min each. The PVDF membrane was placed in a glass dish that contains the secondary antibody (1:1000) and incubated in a shaker for 1 h at room temperature. After the secondary antibody was incubated, it was washed thrice with the TBST buffer for 15 min each. According to the size of the membrane, an appropriate amount of the ECL working fluid was added and allowed to react for several minutes in the dark; a filter paper absorbed the excess liquid. In the gel imaging analyzer, a luminescent signal was acquired, and the image was collected. The gray value of each sample in the protein expression band was quantitatively analyzed by the Image J software.

### 2.7 Statistical analysis

The SPSS 22.0 software was used to process and analyze the experimental data. The data were expressed as means ± SE. Duncan's multiple range test was used for multiple comparisons based on one‐way ANOVA. The relative expression of the target genes was calculated by the 2^−ΔΔCt^ method, *p* < 0.05 indicated significant differences in the results.

## 3 RESULTS

### 3.1 Growth performance and feed utilization

Data related to growth performance and feed utilization are presented in Table 3. The final body weight (FBW) and specific growth rate (SGR) in the HC group were significantly lower than those in the LC group (*p* < 0.05). Compared with the HC group, the FBW and SGR of the R1 and R2 groups were increased by 5.73% and 4.53% (FBW), and 11.57% and 9.09% (SGR), respectively, the difference was not significant. There was no significant difference in the average feed intake among the experimental groups. The feed conversion rate (FCR) in the HC group was significantly higher than that in the LC group (*p* < 0.05). Compared with the HC group, the FCR of R1 and R2 groups decreased by 11.54% and 12.82%, respectively; the difference was not significant. There was no significant difference in the PER between the HC and LC groups, the protein efficiency ratio (PER) in the R1 and R2 groups was significantly higher than that in the HC group (*p* < 0.05).

**TABLE 3:** Effects of rosiglitazone on the growth performance of tilapia.

Items	LC	HC	Rosiglitazone level (mg/kg)		
			R1 (10)	R2 (20)	R3 (30)
IBW^1^	81.21 ± 1.27	80.15 ± 1.42	79.02 ± 0.76	79.33 ± 0.44	79.28 ± 0.51
FBW^2^	161.64 ± 5.14^b^	146.67 ± 4.89^a^	155.07 ± 6.05^ab^	153.32 ± 5.76^ab^	148.36 ± 5.23^a^
SGR^3^	1.38 ± 0.09^b^	1.21 ± 0.02^a^	1.35 ± 0.12^ab^	1.32 ± 0.10^ab^	1.25 ± 0.07^a^
FCR^4^	1.26 ± 0.11^a^	1.56 ± 0.07^b^	1.38 ± 0.18^ab^	1.36 ± 0.13^ab^	1.49 ± 0.12^b^
FI^5^	101.35 ± 3.24	103.74 ± 3.98	104.95 ± 2.55	100.63 ± 2.81	102.93 ± 5.16
PER^6^	2.81 ± 0.16^ab^	2.67 ± 0.09^a^	3.02 ± 0.11^b^	3.05 ± 0.14^b^	2.62 ± 0.15^a^

Data represent means ± SE (n = 5). Values with different letters are significantly different (*p* < 0.05). Absence of letters indicates no significant difference between treatments.

LC, Low starch group; HC, High starch group; R1, high starch + rosiglitazone (10 mg/kg) group; R2, high starch + rosiglitazone (20 mg/kg) group; R3, high starch + rosiglitazone (30 mg/kg) group.

^1^ IBW: Initial body weight (g).

^2^ FBW: Final body weight (g).

^3^ SGR: Specific growth rate (SGR %/d) = 100 × [(Ln (final body weight) − Ln (initial body weight))/duration (50 days)].

^4^ FCR: Feed conversion rate (FCR) = feed intake/(final body weight − initial body weight).

^5^ FI: Feed intake (g).

^6^ PER: Protein efficiency ratio (PER) = live weight gain (g)/dry protein intake (g).

### 3.2 Whole fish body composition

The whole fish body components in all the groups at the end of the feeding trial are presented in Table 4. The crude protein content of the R1, R2, and R3 groups was significantly lower than that of the LC group (*p* < 0.05). However, no significant differences were observed among the R1, R2, R3, and HC groups (*p* > 0.05). No significant difference in the crude lipid content was found among the groups. However, the crude lipid content of the rosiglitazone group was lower than that of the HC group (*p* > 0.05).

**TABLE 4:** Effects of rosiglitazone on whole fish body composition of GIFT tilapia^1^.

Items	LC	HC	Rosiglitazone level (mg/kg)		
			R1 (10)	R2 (20)	R3 (30)
Crude protein **(**%)	16.47 ± 0.54^b^	15.01 ± 0.62^a^	15.13 ± 0.71^a^	15.04 ± 0.73^a^	15.15 ± 0.49^a^
Crude lipid **(**%)	6.79 ± 0.33	7.23 ± 0.41	7.14 ± 0.29	7.11 ± 0.16	6.84 ± 0.32
Ash **(**%)	4.15 ± 0.17	4.13 ± 0.15	4.14 ± 0.12	4.02 ± 0.21	4.24 ± 0.19
Moisture **(**%)	75.88 ± 3.11	75.53 ± 2.26	75.23 ± 2.62	75.70 ± 3.17	75.89 ± 2.11

^1^ Data represent means ± SE (n = 5). Values with different letters are significantly different (*p* < 0.05). Absence of letters indicates no significant difference between treatments.

### 3.3 Serum biochemical parameters

The serum biochemical parameters of all groups are presented in Table 5. The TG content in the HC, R1, R2, and R3 groups was significantly higher than that in the LC group (*p* < 0.05). Meanwhile, the TG content in the R1, R2, and R3 groups was lower than that in the HC group, but the difference was not significant (*p* > 0.05). The TC content in the HC, R1, R2, and R3 groups was significantly higher than that in the LC group (*p* < 0.05). Meanwhile, the TC content in R1, R2, and R3 groups was significantly lower than that in the HC group (*p* < 0.05). The HDL‐C content in the HC, R1, R2, and R3 groups was significantly higher than that in the LC group (*p* < 0.05); the HDL‐C content in the R1, R2, and R3 groups was significantly higher than that in the HC group (*p* < 0.05). Meanwhile, with an increase in the concentration of rosiglitazone, the HDL‐C content increased. The GLB content in the LC, R2, and R3 groups was significantly higher than that in the HC group (*p* < 0.05); the GLB content in the R2 group was significantly higher than that in the LC group (*p* < 0.05). The contents of GOT and GPT in the R1 and R2 groups were significantly lower than those in the HC group (*p* < 0.05). However, the contents of GOT and GPT in the R3 group were significantly higher than those in the R1 and R2 groups (*p* < 0.05).

**TABLE 5:** Effects of rosiglitazone on serum biochemical parameters of GIFT tilapia^1^.

Items	LC	HC	Rosiglitazone level (mg/kg)		
			R1 (10)	R2 (20)	R3 (30)
Triglyceride (TG, mmol/L)	2.56 ± 0.28^a^	3.41 ± 0.26^b^	3.14 ± 0.14^b^	3.10 ± 0.07^b^	3.19 ± 0.18^b^
Total cholesterol (TC, mmol/L)	3.83 ± 0.11^a^	5.27 ± 0.23^c^	4.77 ± 0.04^b^	4.85 ± 0.12b^b^	4.83 ± 0.09^b^
High‐density lipoprotein cholesterol (HDL‐C, mmol/L)	1.02 ± 0.14^a^	1.14 ± 0.16^b^	1.17 ± 0.27^b^	1.29 ± 0.11^b^	1.34 ± 0.15^b^
Low‐density lipoprotein cholesterol (LDL‐C, mmol/L)	0.70 ± 0.09	0.84 ± 0.08	0.78 ± 0.09	0.79 ± 0.05	0.80 ± 0.12
Albumin (ALB, g/L)	13.60 ± 0.56	12.31 ± 0.86	12.33 ± 0.21	12.25 ± 0.63	13.24 ± 0.42
Globulin (GLB, g/L)	21.35 ± 0.35^b^	18.45 ± 0.49^a^	19.33 ± 0.50^a^	22.90 ± 0.41^c^	20.53 ± 0.58^b^
Glutamic oxaloacetic transaminase (GOT, u/L)	40.33 ± 2.08^ab^	42.51 ± 2.23^b^	38.33 ± 1.15^a^	38.01 ± 2.02^a^	42.66 ± 2.51^b^
Glutamic pyruvic transaminase (GPT, u/L)	15.33 ± 0.52^b^	16.02 ± 0.63^b^	12.03 ± 0.74^a^	13.02 ± 0.41^a^	22.01 ± 1.41^c^

^1^ Data represent means ± SE (n = 5). Values with different letters are significantly different (*p* < 0.05). Absence of letters indicates no significant difference between treatments.

### 3.4 Serum glucose and insulin and liver muscle glycogen parameters

**FIGURE 1:**
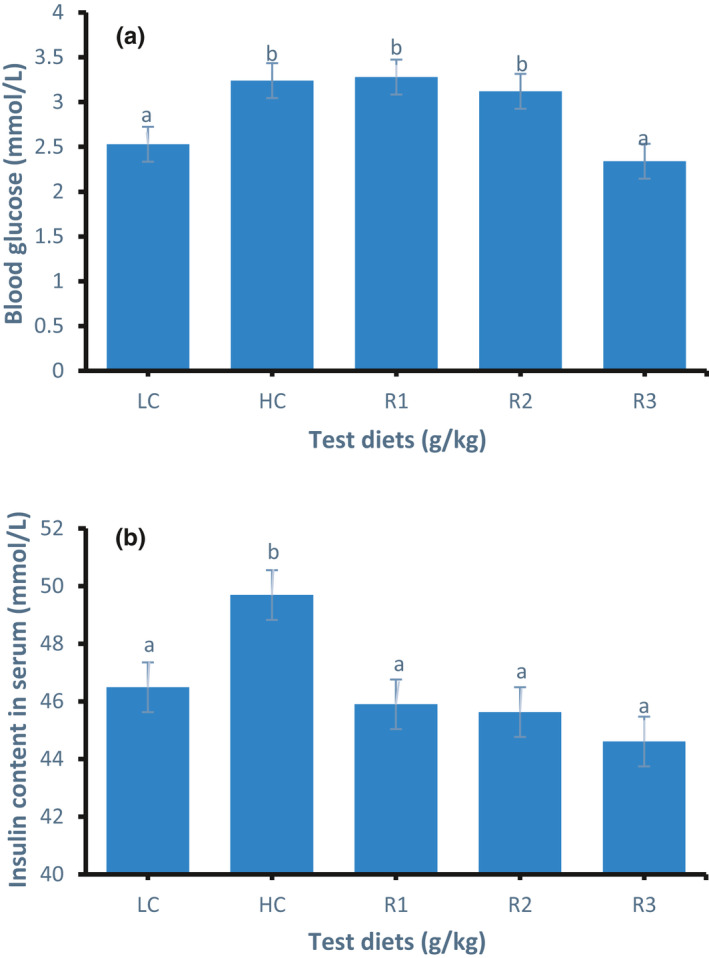
Serum glucose (a) and insulin (b) content in GIFT tilapia fed test diets for 50 days. Data represent means ± SE (N = 5). Values with different letters are significantly different (*p* < 0.05).

The serum glucose parameters of all groups are presented in Figure 1a. The serum glucose content of the HC group increased significantly by 28.06% (*p* < 0.05) compared to the LC group. The serum glucose content of the R3 group significantly decreased (*p* < 0.05) by 27.8% than that of the HC group. With an increase in the rosiglitazone concentration, the serum glucose content of the R1, R2, and R3 groups decreased gradually.

Serum insulin content in all groups is shown in Figure 1b. The insulin content in the HC group was significantly higher (*p* < 0.05) than that in the LC group. Compared with the HC group, the insulin content in the R1, R2, and R3 groups significantly decreased by 7.62%, 8.17%, and 10.22%, respectively (*p* < 0.05). The results showed that rosiglitazone could effectively improve the insulin sensitivity of tilapia.

**FIGURE 2:**
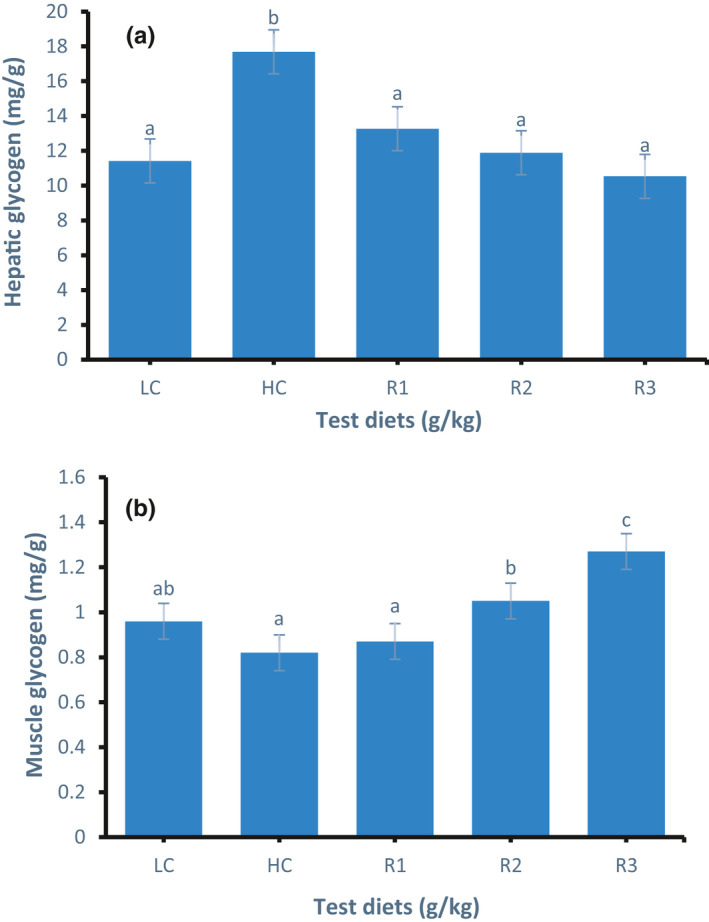
Glycogen content in the liver (a) and muscle (b) of GIFT tilapia fed test diets for 50 days. Data represent means ± SE (N = 5). Values with different letters are significantly different (*p* < 0.05).

Hepatic glycogen content in all groups is presented in Figure 2a. The hepatic glycogen content in the HC group was significantly increased (*p* < 0.05) by 54.9% compared to that of the LC group. Compared with the HC group, the hepatic glycogen content in the R1, R2 and R3 groups decreased significantly (*p* < 0.05); the hepatic glycogen content in the R3 group was the lowest. With an increase in the rosiglitazone concentration, the content of hepatic glycogen in the R1, R2, and R3 groups decreased gradually.

Muscle glycogen content in all groups is shown in Figure 2b. The muscle glycogen content in the R3 group significantly increased (*p* < 0.05) by 32.29% compared with that of the LC group. Compared with the HC group, the muscle glycogen content of the R1, R2, and R3 groups increased gradually with an increase in the rosiglitazone concentration; the muscle glycogen content of the R3 group was the highest.

### 3.5 Gene expression of IRS‐1, PI3K, GLUT‐4, Akt, and GSK

**FIGURE 3:**
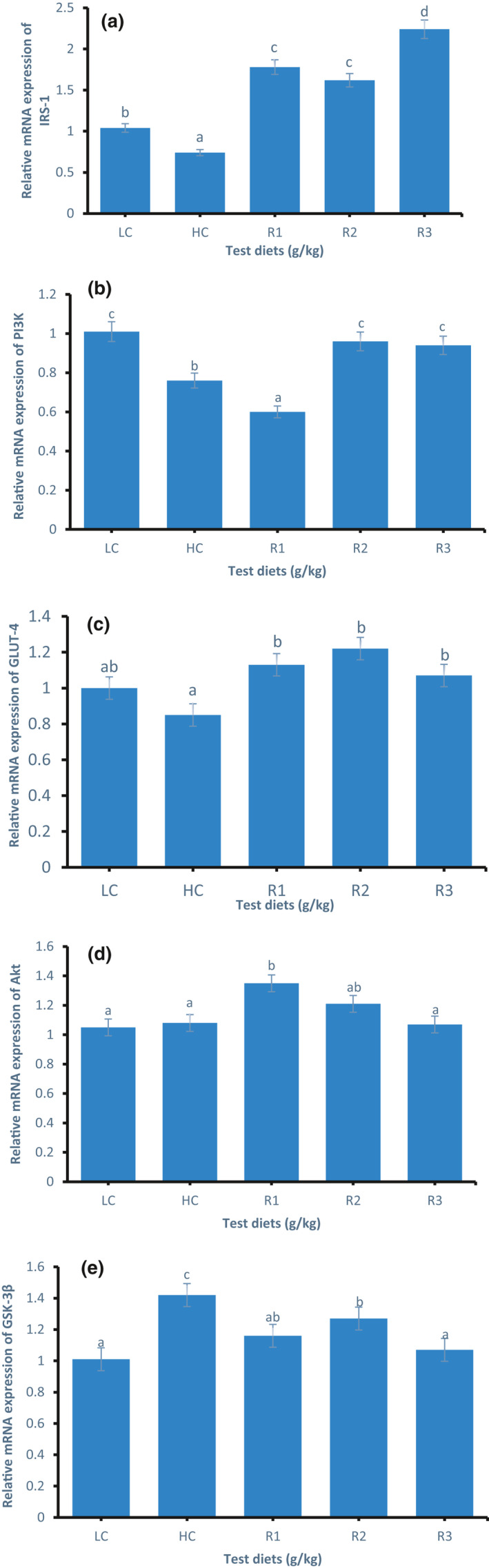
The relative mRNA expression of IRS‐1 (a), PI3K (b), GLUT‐4 (c), Akt (d), and GSK‐3β (e) in the muscle of GIFT tilapia fed test diets for 50 days. The relative mRNA expression was evaluated by real‐time quantitative PCR. Data represent means ± SE (N = 5). Values with different letters are significantly different (*p* < 0.05).

The relative mRNA expression of IRS‐1 in the muscle of GIFT tilapia is shown in Figure 3a. The expression of the IRS‐1 mRNA in the R1, R2, and R3 groups was significantly higher than that in the LC and HC groups (*p* < 0.05); it was the highest in the R3 group. Also the expression of the IRS‐1 mRNA in the HC group was significantly lower than that in the LC group (*p* < 0.05).

The relative mRNA expression of PI3K in the muscle of GIFT tilapia is presented in Figure 3b. The expression of the PI3K mRNA in the HC group was significantly lower (*p* < 0.05) than that in the LC group. Also the expression of the PI3 K mRNA in the R1 group was significantly lower than that in the HC group. However, the expression of the PI3K mRNA in the R2 and R3 groups was significantly higher than that in the HC group (*p* < 0.05).

The relative mRNA expression of GLUT‐4 in the muscle of GIFT tilapia is presented in Figure 3c. The expression of the GLUT‐4 mRNA in the HC group was decreased in comparison with that of the LC group, but the difference was not significant (*p* > 0.05). The LUT‐4 mRNA expression in the R1, R2, and R3 groups significantly increased (*p* < 0.05) in comparison with that of the HC group.

The relative mRNA expression of Akt in the muscle of GIFT tilapia is shown in Figure 3d. No significant difference was observed in the Akt mRNA expression between the R2 and R3 groups (*p* > 0.05) when compared with the LC and HC groups. The expression of the Akt mRNA in the R1 and R2 groups increased with the addition of rosiglitazone in comparison with that of the HC group. Moreover, the expression of the Akt mRNA in the R1 group was significantly higher than that in the HC group (*p* < 0.05).

The relative mRNA expression of GSK‐3β in the muscle of GIFT tilapia is presented in Figure 3e. The expression of the GSK‐3β mRNA in the HC group was significantly higher (*p* < 0.05) than that of the LC group. The expression of the GSK‐3β mRNA in the R1, R2, and R3 groups was significantly lower (*p* < 0.05) than that in the HC group; the expression of the GSK‐3β mRNA in the R3 group was the lowest.

### 3.6 Western blot results for protein expression of p‐Akt and p‐GSK‐3β

**FIGURE 4:**
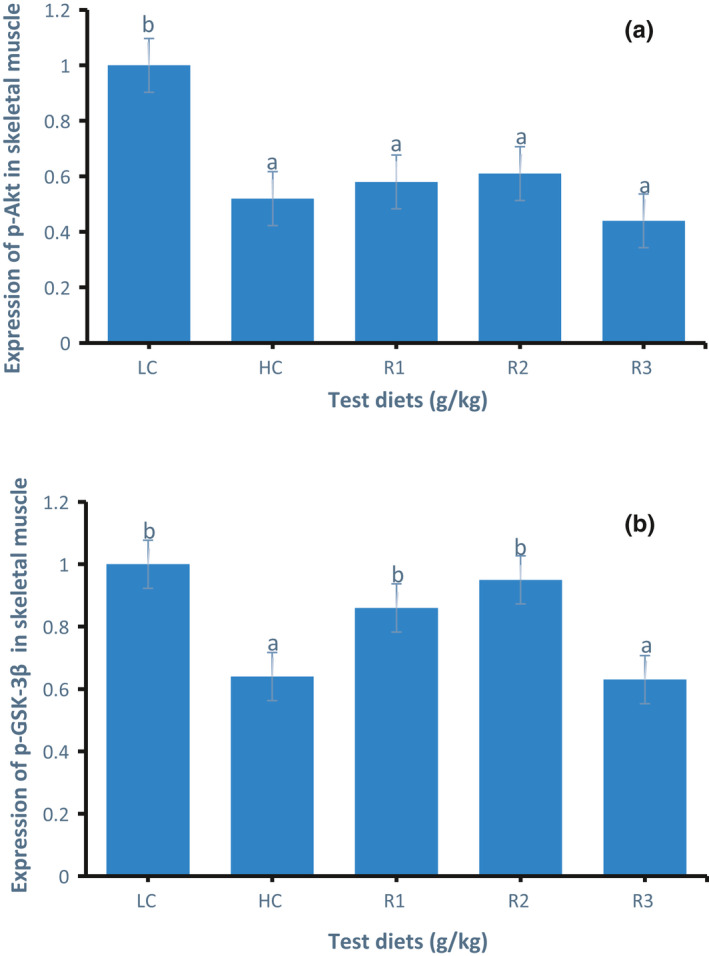
The protein expression of p‐Akt (a) and p‐GSK‐3β (b) in the muscle of GIFT tilapia fed test diets for 50 days. Data represent means ± SE (N = 5). Values with different letters are significantly different (*p* < 0.05).

The protein expression of p‐Akt in the muscle of GIFT tilapia is shown in Figure 4a. The protein expression of p‐Akt in the HC group was significantly lower than that in the LC group (*p* < 0.05). The protein expression of p‐Akt in the R1 and R2 groups was higher than that in the HC group, but the difference was not significant (*p* > 0.05).

The protein expression of p‐GSK‐3β in the muscle of GIFT tilapia is shown in Figure 4b. The protein expression of p‐GSK‐3β in the HC group was significantly lower (*p* < 0.05) than that of the LC group. The protein expression of p‐GSK‐3β in the R1 and R2 groups was significantly higher than that in the HC group (*p* < 0.05). However, no significant difference was observed among the R3 and HC groups (*p* > 0.05).

## 4 DISCUSSION

Presently, studies on rosiglitazone, a regulator of glucose metabolism, are mostly reported in humans and rats; the report on fish is rare. Carey et al. (2000) reported an increase in the subcutaneous fat and body weight of diabetic patients when they took 4 mg of rosiglitazone twice a day for 14 weeks. To study the application of this regulator and improve the carbohydrate utilization in the fish feed, the GIFT tilapia were fed a high starch diet (HC, 53%) supplemented with rosiglitazone in the concentrations of 10, 20, and 30 mg/kg, respectively. The results showed that the growth performance was higher in the groups fed 10 and 20 mg/kg of rosiglitazone compared with that of the high starch group, but this difference was not significant. Meanwhile, the protein efficiency rate (PER) of the 10 and 20 mg/kg groups was significantly higher than that of the high starch group. However, the SGR and PER were not affected when the dosage was 30 mg/kg. These results are similar to those obtained by Yang et al. (2009), wherein they found an increased weight gain rate and significantly reduced feed coefficient after the administration of 20 mg/kg rosiglitazone to *Carassius auratus gibelio*. Zheng et al. (2017) added 0.03% rosiglitazone to the diets that contain normal glucose level (15.6%) and high glucose (30.0%). The results showed that rosiglitazone increased the sensitivity of the insulin receptor to glucose stimulation in the Japanese yellow croaker, but did not significantly affect the growth performance. Zhang et al. (2012) supplemented the high starch diet of *Micropterus salmoides* with 0.3, 1.5, 7.5, and 37.5 mg/kg of rosiglitazone. The results showed that rosiglitazone could prevent the disorder of glucose and lipid metabolism and the occurrence of fatty liver, but it had no significant effect on the growth performance of *Micropterus salmoides*. Shi et al. (2018) found that dietary rosiglitazone (8 mg/d) did not affect the performance of sheep. The differences in the results may be due to the type, specification, diet type, and the amount of rosiglitazone supplemented in the diet. Moreover, it may be due to the dissolution of some other sugar metabolism modulators in the surrounding water that may reduce the final dosage of rosiglitazone. It may also be due to the individuality of fish sugar metabolism. The present results showed that the utilization of carbohydrates in the feed could be improved to some extent by adding rosiglitazone to a high energy diet.

Previous studies on *Larimichthys crocea* (Ma et al., 2017), *Cyprinus carpio* (Keshavanath et al., 2002), and the African catfish (Ali & Jauncey, 2004) found that a high carbohydrate level in the diet had no significant effect on the contents of crude protein, moisture, and ash, but could significantly improve the crude lipid level in the fish. Zhang et al. (2012) found that adding rosiglitazone to the basal diet could effectively reduce the lipid content of *Micropterus salmoides*. Shi et al. (2017) found that the addition of sycamore seed oil and rosiglitazone in diets had no significant effects on production performance, slaughter performance, and meat productivity of sheep. In the current experiment, rosiglitazone had no significant effect on the contents of moisture, ash, crude lipid, and crude protein in the whole body of tilapia. However, the crude fat content decreased with an increase in the rosiglitazone concentration compared with the high starch group, but the difference was not significant. Further, the crude protein content increased with an increase in the rosiglitazone dosage. It was suggested that rosiglitazone increased the sensitivity of the insulin receptor, improved the utilization rate of carbohydrates, reduced the consumption of protein to a certain extent, and increased the protein content in fish. However, Zheng et al. (2017) found that adding 0.03% rosiglitazone to a high sugar diet (30.0%) had little effect on the body composition of the Japanese yellow croaker. Very few studies exist, which describe the role of rosiglitazone in GIFT tilapia, therefore, this phenomenon needs further study.

The contents of triglyceride and total cholesterol in the serum can reflect the status of fat metabolism (Hossain et al., 2016). Rosiglitazone is the ligand for the peroxisome proliferator‐activated receptor γ (PPAR γ) that induces the expression of apolipoprotein and fatty acid oxidase system in the hepatocytes. It increases HDL‐C, decreases LDL‐C and TG, and corrects the disorder in the lipid metabolism (Ahluwalia et al., 2002; Guan et al., 2002). In tilapia, the TG content of the R1 and R2 groups, was 7.92% and 9.09%, respectively, and was lower than that of the HC group, but the difference was not significant. Rosiglitazone also did not significantly affect the contents of HDL‐C and LDL‐C. Yang et al. (2009) also found no significant effect of rosiglitazone on the TG content. Moreover, Yan et al. (2014) found that rosiglitazone decreased levels of serum lipids and fat deposition. The present results showed that rosiglitazone significantly reduced the TC content of tilapia fed the HC diet. However, studies in humans and mice showed that rosiglitazone significantly reduced the contents of TG, TC, and LDL‐C, and significantly increased the level of HDL‐C (Ahluwalia et al., 2002; Guo et al., 2007). The dissimilarity may be due to differences in the species of fish studies or the feeding pattern of the fish. Fish live in water, and some rosiglitazone in the diet may get dissolved in water while feeding; this amount cannot be used by the fish. The total cholesterol and triglycerides in the HC group were significantly higher, indicating that a high amount of carbohydrate in the diet caused sugar intolerance for the fish.

The content of ALB and GLB in the blood can reflect the nutritional status of the animals; an enhanced nutritional status can maintain the content of serum proteins at a high level (levy et al., 1989). The present results showed that there was no significant effect of rosiglitazone on serum ALB level. Yang et al. (2009) also demonstrated that the addition of rosiglitazone did not affect the serum ALB content of *Carassius auratus gibelio*. With tilapia, the level of serum GLB increased initially and then decreased with an increase in rosiglitazone. By supplementing the diet with 20 and 30 mg/kg rosiglitazone, a significant increase was observed in the level of the serum GLB. It has been suggested that rosiglitazone can promote protein synthesis. The GOT and GPT enzymes mainly exist in the hepatocytes and myocardial cells and are important indicators for evaluating the function of the liver and other tissues and organs. The results showed that 10 and 20 mg/kg of rosiglitazone significantly reduced the contents of GOT and GPT in the HC diet. However, when 30 mg/kg rosiglitazone was added, the activity of both serum GOT and GPT increased significantly. This suggested that the excessive addition of rosiglitazone could damage the fish liver. It has also been shown in several human studies that a high dose of RSG can cause liver dysfunction (Freid et al., 2000; Hachey, 2000). In conclusion, dietary rosiglitazone can effectively improve the blood biochemical indices of tilapia, but excessive addition will cause liver damage, and this is not conducive to fish growth.

Rosiglitazone, as an insulin sensitizer, can improve the binding ability of insulin to its receptor and enhance the body's sensitivity to insulin (Guan et al., 2002). It has been widely used in the treatment of human type II diabetes mellitus (Nomura et al., 1999). A large number of studies have shown that rosiglitazone can significantly reduce the fasting blood glucose level and insulin concentration in humans and mice (Stumvol & Haring, 2002). However, supplementing with rosiglitazone in the diet of apolipoprotein E‐deficient mice (1.5 mg/kg) does not affect their blood glucose levels (Li et al., 2019). Yang et al. (2009) found that adding rosiglitazone to the diet of *Carassius auratus gibelio* could significantly reduce the blood glucose and insulin levels. However, Kim et al. (2008) found that rosiglitazone could stimulate the release and synthesis of insulin through the upregulation of GLUT‐2 gene expression. Our results showed that 30 mg/kg rosiglitazone significantly reduced the blood glucose and insulin concentration in the HC group. This observation is consistent with the results in humans, mice, and *Allogynogenetic* crucian carp. Thus the regulatory mechanism of rosiglitazone in the fish may be similar to that in humans and rodents.

Glycogen is mainly stored in the liver and muscle. A certain amount of liver glycogen can regulate uncontrolled blood glucose after a carbohydrate feed. However, the accumulation of excess liver glycogen will cause liver damage in the fish (Hemre et al., 2002). The results showed that rosiglitazone could significantly reduce the content of liver glycogen and alleviate the damage likely to be caused by high glucose to the fish liver. Rosiglitazone (RSG) is a highly selective agonist of peroxisome proliferator‐activated receptor. By activating PPAR, RSG can enhance the inhibitory effect of insulin on hepatic glycogenesis and promote the absorption and utilization of glucose by muscle cells (Lebovitz et al., 2001). In this experiment, the content of muscle glycogen increased gradually with an increase in rosiglitazone. This may be due to the enhancement of glucose absorption in the muscle cells by rosiglitazone.

IRS‐1, PI3K, Akt, GLUT‐4, and GSK‐3β are important regulators in the signaling pathway of glucose metabolism (Wang et al., 2007). The insulin substrate receptor‐1 (IRS‐1) is one of the earliest discovered insulin receptor substrates and is closely related to glucose metabolism (Wilailak et al., 2001). Akt is an insulin receptor and also a downstream signaling molecule of PI3 K. It plays an important role in insulin metabolism, signal transduction, and glucose transport. GSK‐3β is a key enzyme in the glycogen synthesis pathway. Overexpression and overactivation of GSK‐3β in the skeletal muscle tissue are related with the formation of insulin resistance (Wu & Wang, 2018). Rosiglitazone belongs to the class of thiazolidinediones. It can regulate transcription by activating PPAR. Thiazolidinediones can prevent or reverse the toxic effect of hyperglycemia on tyrosine kinase, promote the phosphorylation of IRS‐1 (Shen et al., 2004), increase the level of GLUT‐4 mRNA and protein in the skeletal muscle of insulin‐resistant mice, and promote the translocation of GLUT‐4 in the skeletal muscle (Yonemitsu et al., 2001). In the study on human type Ⅱ diabetes, RSG improved glucose utilization, promoted the expression of IRS‐1 and GLUT‐4, and improved insulin sensitivity (Kirsti et al., 2002; Souza et al., 2003). Moreover, Sundaresan et al. (2016) results strongly suggest that ursolic acid and rosiglitazone combination activates IRS‐PI3‐kinase‐Akt‐dependent signaling pathways to induce GLUT‐4 translocation and increases the expression of insulin receptor to improve glucose intolerance. In tilapia, the expression of IRS‐1 and GLUT‐4 mRNA was significantly increased by adding an appropriate amount of rosiglitazone, and the expression of GLUT‐4 was enhanced by supplementing the diet with 20 mg/kg rosiglitazone. Miyazaki et al. (2000) found that rosiglitazone increased the expression of PI3K in the adipocytes. In this experiment, we also found that RSG increased the expression of PI3K mRNA, but the difference was not significant.

Akt is the main downstream signaling molecule after PI3K activation and a substrate of PDK1. PDK1 phosphorylates Thr308 and Ser437 to activate Akt (Okamoto et al., 2002). The activated Akt returns to the cytoplasm and gets accumulated. It then inactivates GSK‐3β by phosphorylating its Ser9 residue and thus prevents the phosphorylation of GSK‐3β to its substrate GS. Therefore, Akt plays an important role in glycogen synthesis and achieves the goal of lowering the blood glucose level. The results of the Western blot showed that the phosphorylation levels of Akt in the high starch diet and rosiglitazone groups were significantly lower than those in the LC group. Moreover, the Akt phosphorylation level of the 10 and 20 mg/kg rosiglitazone groups was higher than that of the HC group, although the difference was not significant. This indicated that rosiglitazone could promote the phosphorylation of Akt to a certain extent. The present results also showed that supplementation with 10 or 20 mg/kg rosiglitazone could significantly increase the phosphorylation level of GSK‐3β. This helped prevent GSK‐3β from phosphorylating GS and play a role in glycogen synthesis to achieve the goal of lowering blood glucose. This is consistent with the results of our determination of GSK‐3β levels by qPCR. The present results also showed that the phosphorylation levels of Akt and GSK‐3β began to decrease when the dosage was 30 mg/kg. Therefore, a higher concentration of rosiglitazone does not necessarily imply better results; RSG is required in appropriate amounts. In mammalian and mice studies, it was found that p‐Akt activated p‐GSK‐3β and inactivated GSK‐3β, thus preventing GSK‐3β from phosphorylating GS. Thus improved muscle glycogen synthesis and lowered the blood glucose level. The present results on tilapia showed that rosiglitazone in the HC diet could promote the phosphorylation of Akt and GSK‐3β, promote the synthesis of liver glycogen, reduce the level of blood glucose, and also alleviate the liver insulin resistance.

## 5 CONCLUSION

A high starch diet supplemented with rosiglitazone can improve the growth performance, reduce the serum glucose and insulin levels, improve the blood biochemical indices, and increase the muscle glycogen content. It upregulates the mRNA levels of IRS‐1, PI3K, and GLUT‐4 in the skeletal muscle, increases the insulin sensitivity, and promotes glucose uptake. Meanwhile, the phosphorylation of Akt and GSK‐3β significantly increased, resulting in the inactivation of GSK‐3β and the alleviation of insulin resistance.

## CONFLICTS OF INTEREST

The authors declare that they have no conflict of interest.

## AUTHOR CONTRIBUTIONS

Dong‐Yan Guan and Hui‐Wen Sun carried out data analysis and manuscript preparation. Ji‐Ting Wang carried out trial design and modified main document. Zhen Wang and Yang Li coordinated fieldwork and sample collection. Hao‐Jun Han carried out data analysis. Xiang Li and Ting‐Ting Fang carried out feeding trial.

## Data Availability

The data that support the finding of this study are available within the article.
